# A phase II trial of regorafenib in patients with advanced Ewing sarcoma and related tumors of soft tissue and bone: SARC024 trial results

**DOI:** 10.1002/cam4.5044

**Published:** 2022-08-10

**Authors:** Steven Attia, Vanessa Bolejack, Kristen N. Ganjoo, Suzanne George, Mark Agulnik, Daniel Rushing, Elizabeth T. Loggers, Michael B. Livingston, Jennifer Wright, Sant P. Chawla, Scott H. Okuno, Denise K. Reinke, Richard F. Riedel, Lara E. Davis, Christopher W. Ryan, Robert G. Maki

**Affiliations:** ^1^ Mayo Clinic Jacksonville Jacksonville Florida USA; ^2^ Cancer Research and Biostatistics Seattle Washington USA; ^3^ Stanford Cancer Institute Stanford California USA; ^4^ Dana‐Farber Cancer Institute/Brigham and Women's Hospital Boston Massachusetts USA; ^5^ City of Hope Duarte California USA; ^6^ Indiana University Bloomington Indiana USA; ^7^ Fred Hutchinson Cancer Research Center Seattle Washington USA; ^8^ Levine Cancer Institute Charlotte North Carolina USA; ^9^ Huntsman Cancer Institute Salt Lake City Utah USA; ^10^ Sarcoma Oncology Center Santa Monica California USA; ^11^ Mayo Clinic Rochester Minnesota USA; ^12^ Sarcoma Alliance for Research through Collaboration Ann Arbor Michigan USA; ^13^ Duke Cancer Institute Durham North Carolina USA; ^14^ Oregon Health & Science University Portland Oregon USA; ^15^ Abramson Cancer Center, Perelman School of Medicine University of Pennsylvania Philadelphia Pennsylvania USA; ^16^ Lilly Pharmaceuticals Indianapolis Indiana USA

**Keywords:** (5): *CIC‐DUX4*, clinical trial, Ewing sarcoma, regorafenib

## Abstract

**Background:**

Regorafenib is one of several FDA‐approved cancer therapies targeting multiple tyrosine kinases. However, there are few subtype‐specific data regarding kinase inhibitor activity in sarcomas. We report results of a single arm, phase II trial of regorafenib in advanced Ewing family sarcomas.

**Methods:**

Patients with metastatic Ewing family sarcomas (age ≥ 18, ECOG 0–2, good organ function) who had received at least one line of therapy and experienced progression within 6 months of registration were eligible. Prior kinase inhibitors were not allowed. The initial dose of regorafenib was 160 mg oral days 1–21 of a 28‐day cycle. The primary endpoint was estimating progression‐free rate (PFR) at 8 weeks employing RECIST 1.1.

**Results:**

Thirty patients (median age, 32 years; 33% women [10 patients]; bone primary, 40%; extraskeletal primary, 60%) enrolled at 14 sites. The most common grade 3 or higher toxicities were hypophosphatemia (5 grade 3, 1 grade 4), hypertension (2 grade 3), elevated ALT (2 grade 3). Sixteen patients required dose reductions, most often for hypophosphatemia (*n* = 7 reductions in 6 patients); two stopped regorafenib for toxicity. There was one death unrelated to treatment in the 30‐day post‐study period. Median progression‐free survival (PFS) was 14.8 weeks (95% CI 7.3–15.9); PFR at 8 weeks by Kaplan–Meier analysis was 63% (95% CI 46–81%). The RECIST 1.1 response rate was 10%. Median OS was 53 weeks (95% CI 37–106 weeks).

**Conclusions:**

Regorafenib has modest activity in the Ewing family sarcomas. Toxicity was similar to that seen in approval studies.

## Graphical abstract

This single arm phase II study of regorafenib in adults with advanced Ewing sarcoma met its primary endpoint of 8 week progression‐free survival rate of 73%. The RECIST 1.1 response rate was 10%.

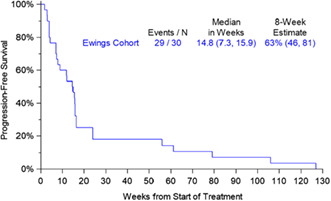

## 1 INTRODUCTION

Ewing sarcoma, or more broadly the Ewing family of tumors, represents the second most common bone sarcoma in children, with a cure rate of 75–80% in children with aggressive multi‐agent chemotherapy, surgery, and often radiation for localized disease. Outcomes for late teenagers and adults are less satisfactory. The primary tumor in Ewing sarcoma more commonly occurs as an extraskeletal mass in adults compared to bone in children.^1^

For patients with relapsed disease, topoisomerase I inhibitor‐based therapy is a standard of care. Two commonly used combinations are irinotecan‐temozolomide^2^, ^3^ or cyclophosphamide‐topotecan, and high‐dose ifosfamide has activity.^4^, ^5^, ^6^ Insulin‐like growth factor 1 receptor inhibitors are associated with a 5%–15% response rate in phase II studies.^7^ Pazopanib and trabectedin are approved in some countries for treatment of sarcoma, but there are few data regarding the specific activity of either agent in the Ewing sarcoma family of tumors.^8^, ^9^ Immune checkpoint inhibitors appear inactive in metastatic/recurrent Ewing sarcoma.^10^

In seeking other systemic therapeutic agents, preclinical^11^, ^12^ data support targeting angiogenesis in Ewing sarcoma. Additionally anecdotes of clinical activity with multi‐targeted receptor tyrosine kinase inhibitors such as pazopanib have been reported in metastatic Ewing sarcoma patients.^13^, ^14^, ^15^, ^16^ Given what was at the time a lack of prospective data,^17^ and in order to determine if an oral kinase inhibitor were active in specific sarcoma subtypes, we designed SARC024, a phase II basket trial of the oral broad spectrum VEGF‐targeted tyrosine kinase inhibitor regorafenib (Bayer; Berlin, Germany) in advanced liposarcoma, osteogenic sarcoma, rhabdomyosarcoma, and Ewing family sarcoma patients. We report here the results from the Ewing family sarcoma cohort of patients, which was the first cohort to complete accrual.

## 2 MATERIALS AND METHODS

### 2.1 Eligibility

Prior to registration, eligible patients had either a diagnosis of Ewing/Ewing‐like sarcoma with classic translocation, Ewing‐like sarcoma with proof of novel translocation such as *CIC‐DUX4*, *BCOR‐CCNB3* or related genes, or histological diagnosis of Ewing sarcoma family of tumors without proof of translocation. Patients with extraskeletal Ewing sarcoma were permitted. Requirements at baseline included age ≥18, WHO performance status ≤2, receipt of a prior line of systemic therapy in the neoadjuvant, adjuvant or advanced setting, measurable disease by Response Evaluation Criteria in Solid Tumors (RECIST) 1.1^18^ with evidence of progressive disease (PD) within 6 months and adequate organ function.

Exclusion criteria included prior small molecule oral kinase inhibitor, clinically significant other active malignancy within 12 months, prior systemic treatment <14 days of registration or toxicities from treatment not recovered to grade ≤1 (except alopecia), investigational therapy <5 half‐lives or <14 days (whichever was greater), major surgery or wide field radiotherapy ≤28 days, limited field palliative radiotherapy <14 days, uncontrolled hypertension, clinically significant cardiac disease, history of bleeding diathesis, bleeding event ≥CTCAE version 4.03 grade 3 within 4 weeks, thromboembolic event within 6 months, HIV or hepatitis B or C infection requiring antiviral therapy, ongoing infection CTCAE version 4.03 Grade 3 or worse, non‐healing wound, seizure disorder requiring medication, proteinuria >100 mg/dl, symptomatic interstitial lung disease, pleural effusion or ascites causing ≥CTCAE v4.03 Grade 2 dyspnea, history of organ allograft, known hypersensitivity to regorafenib, malabsorption condition, or use of an herbal remedy.

### 2.2 Treatment plan

Initial prescription for cycle 1 was oral regorafenib provided as research drug by Bayer at 160 mg (4 × 40 mg tablets) taken in the morning with a low fat meal on days 1–21 of a 28‐day cycle. Unlike the osteosarcoma and liposarcoma cohorts, the Ewing family sarcoma cohort was not placebo‐controlled, given the concern of placing patients with rapidly progressive disease on a placebo. Patients remained on study treatment until either RECIST 1.1^18^ progression, dose interruption of greater than 28 days or patient‐ or physician‐initiated withdrawal. All patients who received at least one dose of regorafenib were deemed eligible for toxicity and response assessment.

### 2.3 Toxicities and dose modifications

Toxicities were graded with Common Toxicity Criteria Adverse Events (CTCAE) version 4.03. Dose reductions were permitted for clinically significant Grade 2 toxicities related to regorafenib at the discretion of the investigator, and required for clinically significant toxicities ≥Grade 3. Up to two dose reductions by 40 mg decrements were permitted (i.e., to 120 and 80 mg, days 1–21 of a 28 cycle).

### 2.4 Response assessment

Tumor assessments were performed utilizing RECIST version 1.1.^18^ The baseline study scan was required within 28 days of cycle 1 day 1. Thereafter, tumor assessments were performed every 8 weeks for the first 32 weeks, then every 12 weeks, with a ±7 day window of the anticipated scan date.

### 2.5 Statistical considerations

The primary endpoint of the study was to estimate the progression‐free rate (PFR) using RECIST 1.1^18^ of eligible patients treated with regorafenib at 8 weeks after starting treatment. Secondary endpoints included response rate (RR), overall progression‐free survival (PFS) and overall survival (OS) for the entire cohort.

Because the median progression‐free survival rate is very short in patients with recurrent/refractory Ewing family sarcomas, even employing agents with modest activity such as IGF1R inhibitors,^7^ an open‐label single‐stage design was employed, using PFS at 8 weeks as a marker of minimal activity. A progression‐free rate (PFR) of 50% at 8 weeks was considered to be necessary to rule out an entirely inactive agent in this cohort, in comparison to an uninteresting PFR at 8 weeks of 25%. For this 30‐patient cohort with these parameters, this yields a false positive rate of 0.05 (alpha) and power (1‐beta) of 0.91. The response rate was estimated and a confidence interval constructed. A sample size of 30 patients permitted a 95% confidence interval within ±18%.

## 3 RESULTS

This study was performed after approval by institutional review boards at participating sites in accordance with an assurance filed with and approved by the U.S. Department of Health and Human Services. The study was registered with ClinicalTrials.gov with identifier NCT02048371. All patients provided written informed consent to participate in this study.

### 3.1 Enrollment

Baseline patient characteristics are shown in Table 1. Notable in the enrolled population was the preponderance of an extraskeletal primary site, which is more typically seen in adults and is distinct from pediatric patients, who typically have bony primary sites of disease. The median time from initial diagnosis to treatment was 41.9 months (range 0.8–233 months). The median time from the diagnosis of either recurrent or metastatic disease to the start of regorafenib was 19.7 months (range 0.5 to 130 months).

**TABLE 1:** Baseline patient characteristics (*n* = 30)

Characteristic	Value
Age (years)	
Median (range)	32 (19–65)
Gender, *n* (%)	
Female	10 (33)
Male	20 (67)
Race, *n* (%)	
White	28 (93)
Asian	1 (3)
Unknown	1 (3)
WHO performance status, *n* (%)	
0	16 (53)
1	13 (43)
2	1 (3)
Primary tumor location, *n* (%)	
Abdomen	1 (3)
Cervix	1 (3)
Extremity	9 (30)
Kidney	1 (3)
Lung	3 (10)
Other	4 (13)
Ovary	1 (3)
Pelvis	5 (17)
Peritoneum	1 (3)
Spinal cord	1 (3)
Spine	2 (7)
Uterus	1 (3)
Primary site type, *n* (%)	
Bone	12 (40)
Extraskeletal	18 (60)
Number of prior lines of systemic therapy, *n* (%)	
1	5 (17)
2	10 (33)
3	5 (17)
4	4 (13)
≥5	6 (20)
Months from diagnosis to start of trial	
Median, range	42, 1–233

### 3.2 Treatment and toxicities

Table 2 shows most common toxicities deemed at least possibly related to regorafenib, which were similar to the known toxicities of regorafenib at this dose and schedule. Most common Grade 3 or worse toxicities included hypophosphatemia (6 patients), hypertension (2) and elevated alanine transaminase (2). One patient each experienced other Grade 3 toxicities including oral mucositis, diarrhea, fatigue, abdominal pain, rash, AST increase, hypokalemia, neutropenia, and increased lipase. One Grade 4 treatment‐related case of hypophosphatemia was noted.

**TABLE 2:** Most common regorafenib‐related treatment toxicities (CTCAE version 4.03), *n* = 30 evaluable patients

Adverse event	CTCAE Severity (number of patients)			
	Grade 1–2	Grade 3	Grade 4	Total
Hypophosphatemia	0	5	1	6
Hypertension	3	2	0	5
Alanine aminotransferase increased	0	2	0	2
Mucositis, oral	7	1	0	8
Diarrhea	3	1	0	4
Fatigue	1	1	0	2
Abdominal pain	1	1	0	2
Rash, maculo‐papular	1	1	0	2
Aspartate aminotransferase increased	1	1	0	2
Hypokalemia	0	1	0	1
Neutrophil count decreased	0	1	0	1
Lipase increased	0	1	0	1
Palmar‐plantar erythrodysesthesia syndrome	13	0	0	13
Nausea/vomiting	7	0	0	7
Voice alteration	3	0	0	3
Headache	3	0	0	3
Blood bilirubin increased	2	0	0	2
Fever	1	0	0	1
Alopecia	1	0	0	1
White blood cell decreased	1	0	0	1
Platelet count decreased	1	0	0	1
Anorexia	1	0	0	1
Back pain	1	0	0	1
Constipation	1	0	0	1
Erythema multiforme	1	0	0	1
Malaise	1	0	0	1
Myalgia	1	0	0	1
Peripheral nerve infection	1	0	0	1
Upper respiratory infection	1	0	0	1
Total	57	18	1	76

Sixteen patients required at least one dose reduction, most commonly related to hypophosphatemia (six unique patients; one patient required two dose reductions). Two of these patients receiving dose reductions permanently discontinued regorafenib for toxicity. There was one patient death in the 30‐day post‐study follow up period, assessed to be unrelated to treatment.

The median dose being taken by patients at time of their discontinuation from study was 120 mg (3 tabs, range 80–160 mg).

### 3.3 Efficacy

Two patients stopped treatment prior to having an on‐treatment scan and were censored at 30 and 62 days, respectively, on the Kaplan–Meier curves: one patient stopped treatment prior to completing cycle 1 due to clinical progression, and another patient stopped treatment before having cycle 2 week 4 scans due to physician‐initiated withdrawal after the patient's condition rendered them unacceptable for further treatment. These patients were included as non‐responders in the calculation of response rate.

**FIGURE 1:**
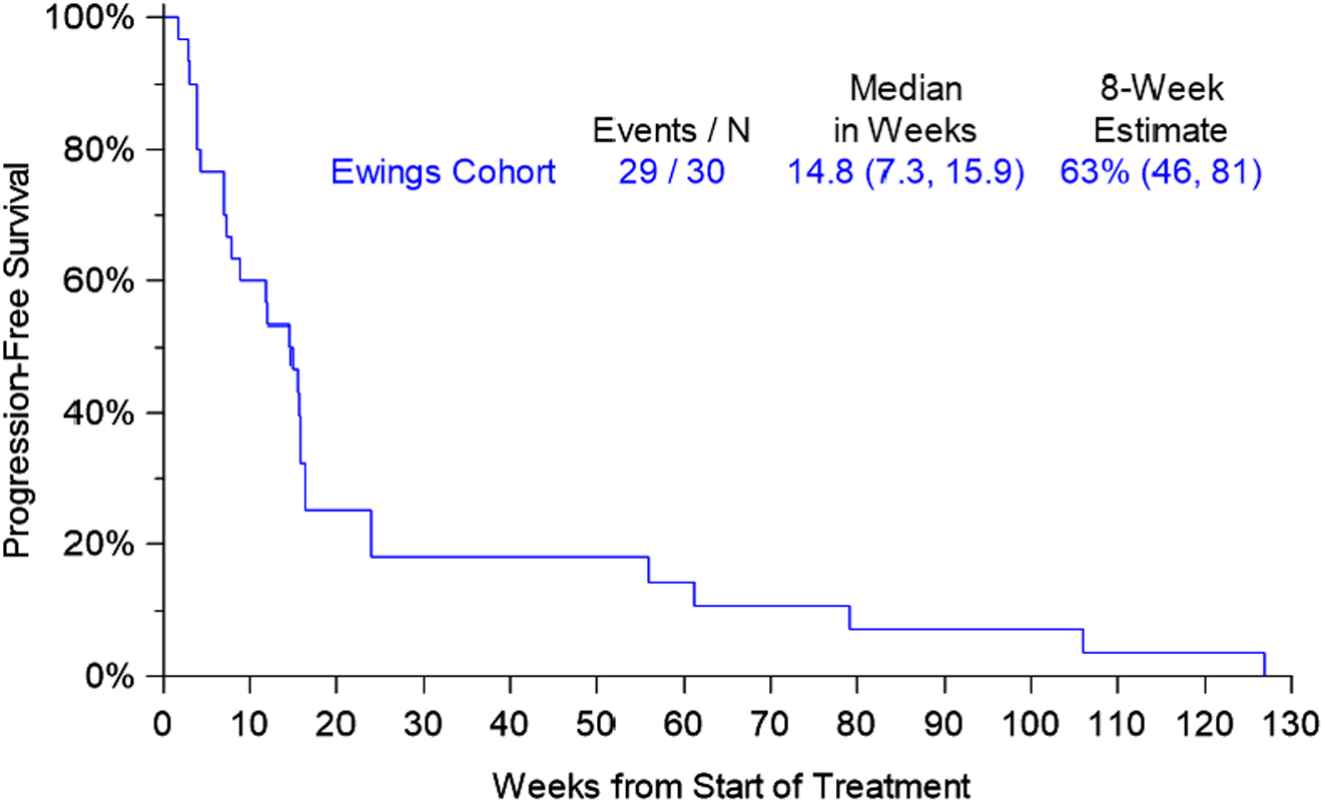
Progression‐free survival by Kaplan–Meier method.

Nineteen of 30 evaluable patients evaluated for PFS were without RECIST 1.1 progression as determined by on treatment scans at 8 weeks. The median PFS was 14.8 weeks (95% CI 7.3, 15.9). The primary endpoint of this study was progression‐free rate at 8 weeks as estimated using the Kaplan–Meier method, which was 63% (95% CI 46–81%) (Figure 1). The PFR at 16 weeks was 36% (95% CI 17–54%) by Kaplan–Meier estimate. Of 18 patients with stable disease as best result by RECIST 1.1, 10 patients (56%) had 3 or more prior lines of therapy. This is similar to the overall study population, where 52% of patients had 3 or more prior lines of therapy.

The RECIST 1.1 response rate was 10% (3/30 patients). All the responding patients had received prior vincristine‐doxorubicin‐cyclophosphamide based therapy. Two patients with partial response (PR) had an *EWSR1* translocation by FISH. The first was a 30‐year‐old woman with a metastatic uterine extraskeletal Ewing sarcoma who received 32 cycles of regorafenib on study and came off treatment due to progressive disease. The second was a 25‐year‐old woman with kidney as her primary tumor site who withdrew consent after 3.5 months of study treatment. This patient's last disease assessment was the date of partial response. One patient with PR had a *CIC‐DUX4* translocation and was also known to have *NRAS* Q61K, *TP53* R282Q, and a *FUS‐ERG* fusion. The patient stopped treatment for PD after 3.5 months. The duration of response for the three patients with PR was 2.93, 27.33, and 0, respectively, due to lack of follow up of the latter patient after withdrawal of consent.

**FIGURE 2:**
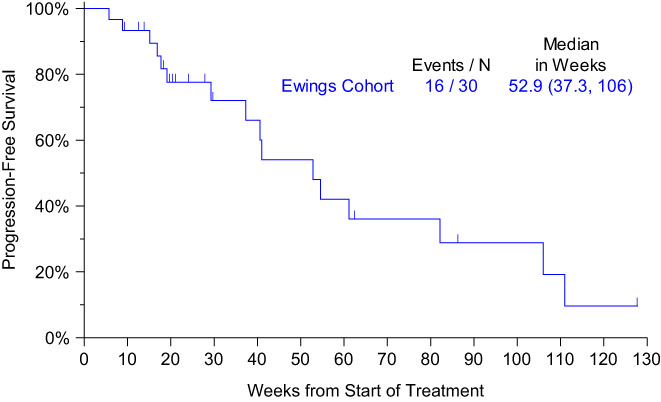
Overall survival by Kaplan–Meier method.

**FIGURE 3:**
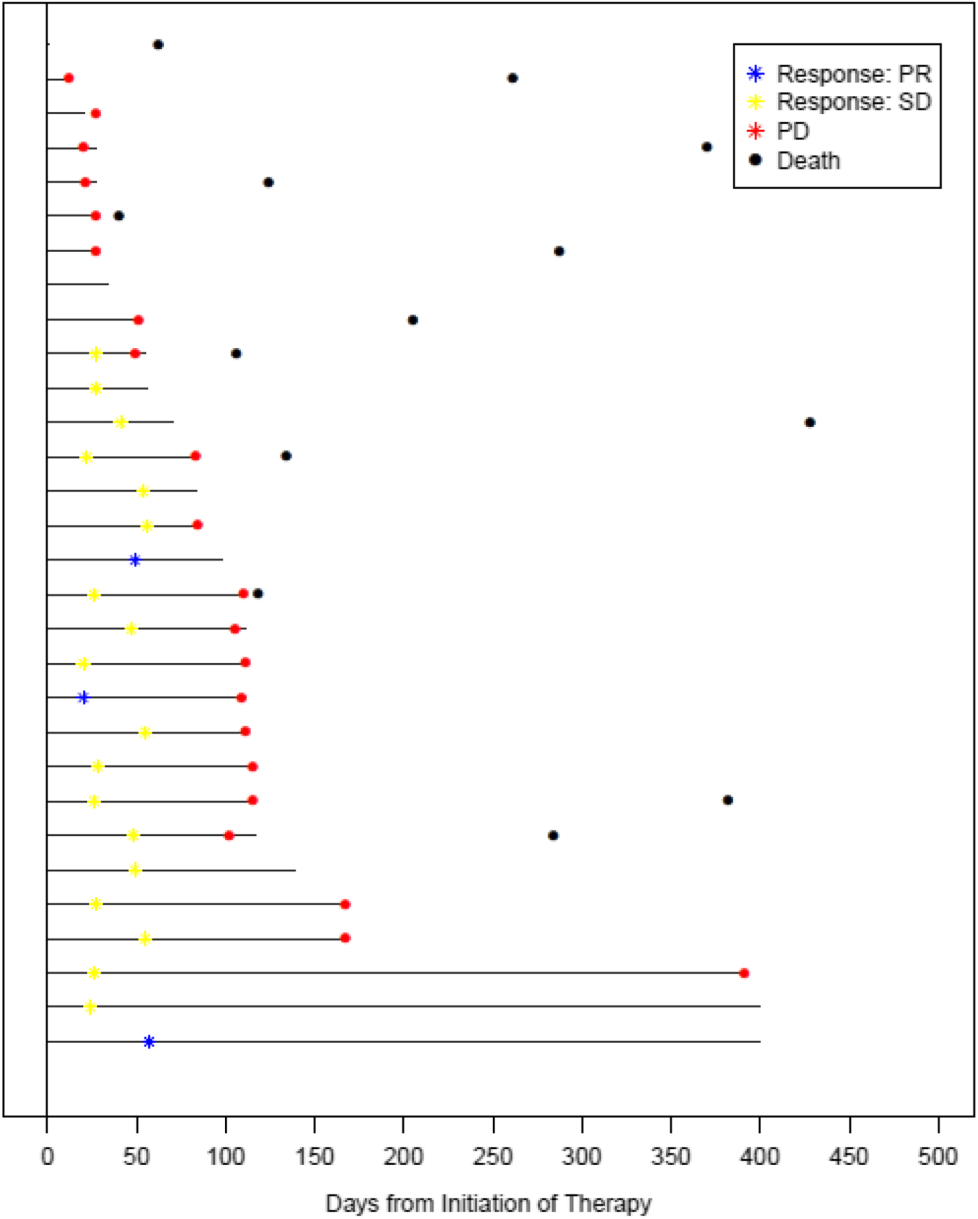
Swimmer's plot of the duration of treatment with regorafenib for the Ewing family sarcoma patients treated on SARC024.

As of 8/2018, median overall survival for the group of 30 patients was 53 weeks (95% CI 37–106 weeks) (Figure 2). No further comprehensive outcomes data were available after this data cut. A swimmer's plot is provided to indicate duration of treatment and outcomes for the patients enrolled on this study. (Figure 3).

## 4 DISCUSSION

When the trial was designed, IGF1R inhibitors had shown a 5%–15% response rate in Ewing sarcoma, but a short median PFR, for example, 7.5 weeks in a Ewing sarcoma subset of a phase II trial of cixutumumab and temsirolimus in metastatic sarcomas.^19^ We used the data from phase II trials of IGF1R inhibitors as a starting point to define a minimum criterion for activity of regorafenib in metastatic Ewing family sarcoma patients, who most commonly had received two or more prior lines of therapy.

Regorafenib at standard doses in this population of patients with metastatic Ewing‐family sarcomas led to a median PFS of 16 weeks (3.7 months), and a progression‐free rate of 73% at 8 weeks, and therefore the study met its primary endpoint. Three partial responses were observed for a RECIST 1.1 response rate of 10%. Notably, one of these responders received treatment for longer than 1 year, and two patients were on study treatment with stable disease for 12 and 18 months. These data support and are consistent with the REBOGONE trial, in which Ewing sarcoma patients treated with regorafenib experienced 2.6 month median PFS (11.4 weeks) for Ewing sarcoma patients versus 0.9 months for placebo.^17^

In comparison, pazopanib received regulatory approval for treatment of all soft tissue sarcomas on the basis of the PALETTE study, based on a 4.6 month median PFS and a response rate of 6%.^20^ These criteria also support the idea that regorafenib merits further study in Ewing sarcoma, given the recognized aggressiveness of Ewing sarcoma compared to other sarcomas of soft tissue and bone. What is genuinely active therapy remains to be determined. For example, gemcitabine‐docetaxel is considered to have at most minor activity in Ewing sarcoma, with a median PFS of 3.0 months in the rEECur trial of relapsed Ewing sarcoma patients randomized to different systemic therapies, which is similar to the outcomes from this trial.^21^ We also note the important difference between a response defined by scans alone and that experienced by the patient, who experiences clinical toxicity that tempers any progression‐free survival benefit. This study did not collect patient‐reported outcomes data, which are increasingly recognized as important to consider as endpoints in cancer clinical trials.^22^

The interpretation of this arm of the SARC024 trial is made more complex due to the inclusion of patients with both Ewing sarcoma and Ewing‐like sarcomas. The latter sarcomas are defined by translocations other than the canonical *EWSR1‐FLI1*, (e.g. *BCOR‐CCNB3* and *CIC‐DUX4*). Ewing‐like sarcomas are characterized by different responsiveness to standard therapy than Ewing sarcoma, with superior outcomes in the case of *BCOR‐CCNB3* tumors and inferior outcomes with *CIC‐DUX4* sarcomas.^23^, ^24^ The finding of a responding patient with a *CIC‐DUX4* translocation raises interesting questions as to the nature of the apparent kinase dependency in these types of sarcoma that may be different from Ewing sarcoma per se.

In Ewing sarcoma, as with Ewing‐like sarcomas, key biological dependencies that might be targeted in combination with regorafenib remain undefined. One possible class of drugs to combine are PARP inhibitors. Elegant biological studies showed poly‐ADP ribose polymerase (PARP) inhibitors were active in Ewing sarcoma cell lines in vitro,^25^ but clinical trials with single agent PARP inhibitors yielded no activity.^26^ A second potential agent to consider in a regorafenib combination is an IGF1R inhibitor. While responses were seen in metastatic Ewing sarcoma using IGF1R inhibitors, Ewing sarcoma patients did not derive benefit from the use of IGF1R inhibitors as part of primary therapy.^27^ As a result, combinations of any agent with regorafenib in Ewing sarcoma should be examined in model systems before moving into clinical trials, especially given many new targets that are emerging in preclinical studies.

We conclude that regorafenib demonstrates modest activity as a single agent against metastatic Ewing family sarcomas, with no new toxicity signal seen in this trial compared to other studies completed in a variety of other cancers. These data support the REGOBONE randomized trial showing activity of regorafenib in Ewing sarcoma in a randomized phase II trial. The biological basis for this result remains elusive. As work in cell lines and other models becomes available, we hope to examine tissue collected from this trial to test hypotheses that may inform future generations of trials in metastatic Ewing and related sarcomas.

## AUTHOR CONTRIBUTIONS

The authors contributed to this manuscript as per the following. SA, SO, DRei, RR, LD, CR, RM: Conceptualization, investigation, methodology, supervision, writing—original draft, and writing—review and editing. VB: Data curation, formal analysis, writing—original draft, and writing—review and editing. KG, SG, MA, DRus, EL, ML, JW, SC: Investigation, writing—original draft, and writing—review and editing.

## FUNDING INFORMATION

Bayer Healthcare.

## CONFLICT OF INTEREST

The following conflicts of interest are reported. *SA*: Research Funding: AB Science, Bayer, Blueprint Medicines, CBA Pharmaceuticals, CytRx, Daichi Sankyo, Deciphera, Desmoid Tumor Research Foundation, Epizyme, Genmab, Gradilis, Immune Design, Incyte, Karyopharm Pharmaceuticals, Lilly, Merck, Morphotek, Novartis, Pilogen, Takeda Oncology, Threshold Pharmaceuticals, Tracon. *SG*: Research Funding: Bayer, Blueprint Genetics, Deciphera, Novartis, Pfizer. Advisory Board: AstraZeneca. Other Interests: Abbvie. *DRei*: Research funding: Bayer. *DRus*: Advisory Board: Lilly. *SC*: Research funding: Amgen, CytRx Corporation, GlaxoSmithKline, Ignyta, Immune Design, Roche, Threshold Pharamceuticals, Tracon. *RR*: Research Funding: AADi, AROG, Ignyta, Immune Design, Karyopharm, Lilly, Limbguard LLC, NanoCarrier, Novartis, Oncternal, Plexxikon, Threshold, Tracon. Advisory Board: Bayer, EISAI, EMD Serono, Janssen, Loxo. *CR*: Research funding: Argos Therapeutics, Bayer, BMS, CytRx, Daiichi Sankyo, Eisai, Exelixis, Genentech, GlaxoSmithKline, Novartis, Janssen, Karyopharm Therapeutics, MabVax, Merck, Morphotek, Threshold Pharmaceuticals, Tracon. *RM*: Research funding: Astex, Bayer, Boeringer Ingleheim, Exelixis, Genentech, Karyopharm, Presage, Rain, Springworks, Synox, Tracon. Consulting/Honoraria: AADi, Bayer, Deciphera, Immune Design, Karyopharm, Presage, Springworks, American Board of Internal Medicine, American Society for Clinical Oncology, UptoDate. Other: Bayer, Tracon. *VB*, *KG*, *EL*, *ML*, *JW*, *SO*, *LD*: No conflicts to report.

## ETHICS STATEMENT

The trial was approved by the Institutional Review Boards of the participating centers and conducted according to the principles of the Declaration of Helsinki and the Guidelines for Good Clinical Practice.

## Data Availability

Data sharing is not applicable to this article as no new data were created or analyzed in this study.
